# Structural and In Vitro Functional Comparability Analysis of Altebrel™, a Proposed Etanercept Biosimilar: Focus on Primary Sequence and Glycosylation

**DOI:** 10.3390/ph12010014

**Published:** 2019-01-17

**Authors:** Ramin Fazel, Yudong Guan, Behrouz Vaziri, Christoph Krisp, Laura Heikaus, Amirhossein Saadati, Siti Nurul Hidayah, Manasi Gaikwad, Hartmut Schlüter

**Affiliations:** 1 Department of Biotechnology, College of Science, The University of Tehran, 1417864311 Tehran, Iran; Ramin.Fazel@gmail.com; 2 Mass Spectrometric Proteomics, Institute of Clinical Chemistry and Laboratory Medicine, Campus Forschung, N27 Raum 00.008, Universitätsklinikum Hamburg-Eppendorf, Martinistr. 52, 20246 Hamburg, Germany; y.guan@uke.de (Y.G.); c.krisp@uke.de (C.K.); l.heikaus@uke.de (L.H.); s.hidayah@uke.de (S.N.H.); m.gaikwad@uke.de (M.G.); 3 Biotechnology Research Center, Pasteur Institute of Iran, 1316943551 Tehran, Iran; Behrouz-vaziri@pasteur.ac.ir; 4 AryoGen Pharmed, Cross Tajbakhsh Street, 24th Kilometer Makhsous, Tehran, Iran; saadatirada@aryogen.com

**Keywords:** biosimilar, etanercept, mass spectrometry, glycosylation, oxidation, amidation, comparability study, biopharmaceutics, permethylation, functional assay

## Abstract

The demand for reliable comparability studies of biosimilars grows with their increased market share. These studies focus on physicochemical, structural, functional and clinical properties to ensure that a biosimilar has no significant differences to the originator product and can be released into the market without extensive clinical trials. In the current study, Enbrel^®^ (etanercept, the originator) and Altebrel™ (the proposed biosimilar) underwent direct comparison. “Bottom-up” mass spectrometric analysis was used for primary sequence analysis, evaluation of N/O-glycosylation sites and quantification of methionine oxidation. N/O-glycans were analyzed after permethylation derivatization and the effect of N-glycans on in-vitro functionality of etanercept was assayed. Three enzyme peptide mapping resulted in complete identification of the primary structure. It was confirmed that total ion chromatograms are valuable datasets for the analysis of the primary structure of biodrugs. New N/O-glycan structures were identified and all the N-glycans were quantified. Finally, investigation of the functional properties of N-deglycosylated and non-modified etanercept samples using surface plasmon resonance analysis and in-vitro bioassay showed that N-glycosylation has no significant effect on its in-vitro functionality. Analysis of etanercept and its biosimilar, revealed a high similarity in terms of glycosylation, primary structure and in-vitro functionality.

## 1. Introduction

In relation to the increased interest of pharmaceutical companies to produce recombinant drugs and their biosimilar versions, the demand for analyzing the physicochemical, structural, functional and clinical properties of biologics has increased. According to current regulations, biosimilar products should show no significant difference to the originator product and therefore clinical Phase 2 trials can be omitted (since the product dosing has already been stablished) whereas Phase 1 and Phase 3 trials still have to be conducted [1]. However, proteins are of a much more complex nature than conventional chemical drugs, complicating the evaluation of their physicochemical and structural properties. Therefore, addressing the regulatory requirements for approval of a biologic or a biosimilar requires state-of-the-art technology and know-how of biopharmaceutical manufacturing processes [2]. Accordingly, this has resulted in the definition of distinct legal and regulatory frameworks in various parts of the world [3]. Although there are many guidelines and publications with an intent to clarify the principles of assessing the comparability of biotechnological products [4,5,6], in many cases the exact strategy and the requirements for the product development or biosimilarity demonstration remain unclear. To meet the standards of drug agency approval and guarantee the quality, safety and efficacy of the drug, thorough research for in-depth characterization of biologics is needed. The comparability studies for each biologic is not based on a “one-size-fits-all” approach and has to be designed specifically. Moreover, the regulatory requirements for manufacturing a recombinant drug are changing, based on new findings making the manufacturing more competitive each day.

Etanercept (Enbrel^®^) is a therapeutic fusion protein composed of human IgG Fc fragment and two extracellular domains of the TNF-α (p75) receptor. The TNF-α receptor contains the functional receptor domain and a highly glycosylated linker connected to the hinge domain of the human IgG Fc containing the CH2 and CH3 domains [7,8]. It is a homodimer connected by disulfide bonds able to block one TNF-α molecule at a time. The complex nature of this fusion protein makes its structural and physicochemical analysis difficult. In a practical view, several sites of N/O-glycosylation along with the other common modifications such as oxidation, deamidation and N/C-terminal heterogeneity makes it a problematic target for physicochemical characterization.

Biosimilars of this biologic have been approved and are being produced in different countries with diverse levels of regulations [9]. Since market is a driving force of pharmaceutical research, vast numbers of case studies have been performed on the blockbuster drug etanercept and each has deciphered different aspects of etanercept structure or function [10]. Since the first biosimilar of etanercept was approved in the European Union (EU) in 2016 [7], several comparability studies have been published aiming to prove the biosimilarity of their product with the originator drug. Jacob et al. [10] and Azevedo et al. [9] have listed the published evidence, which summarizes the comparability studies on etanercept. These publications include clinical and nonclinical studies, randomized control trials, analytical and comparability studies, health economic evaluations and post-marketing studies. Rather than the comparability studies that compared and analyzed the structure and function of a biosimilar with the originator, several publications can also be found which evaluate the different properties of etanercept in detail. These studies included the evaluation of glycosylation sites, composition or functional effects [8,11,12,13,14], analysis of the oxidation or deamidation sites and their effects on function and structure [15,16] as well as disulfide bonds and their functional effects [17]. In this study, the individual mass spectrometric measurements were examined and compared. It has been shown that subtractive TIC analysis can give first hints regarding differences in the composition of the amino acid sequences as well as post-translational modifications.

The combination of a mass spectrometric workflow and the functional assays can offer a unique approach to link observed structural variations to the in-vitro functionality of the biologics. In this regard, comparability analysis of the originator (Enbrel^®^) and a proposed biosimilar (Altebrel™) was performed to investigate the structural and functional variations, which may occur during the recombinant protein manufacturing process. The structure and function of three batches of the etanercept from two vendors were analyzed. The primary structure was inspected using multi-enzyme digestions in the bottom-up approach. There are different methods for carbohydrate sequencing such as utilizing highly specific exoglycosidases in conjunction with the instruments like capillary electrophoresis [18]. In the present study, different N/O-glycosylation sites and compositions were identified and characterized using permethylation as a derivatization technique. Furthermore, a unique strategy was used to evaluate the structure-activity relationship between the N-glycosylation and the biological activity of etanercept by functional assays including SPR affinity analysis and TNF sensitive L929 cell necroptosis neutralization assay. Our findings about the primary sequence and glycosylation, present Altebrel™ as a highly similar form of Enbrel^®^. However, further investigations are needed to declare the similarity in terms of quality, safety and efficacy.

## 2. Materials and Methods

### 2.1. Materials

Three different lots of Enbrel^®^ (G30909, H17609, H42831; Pfizer, Istanbul, Turkey) and Altebrel™ (9202006, 9202008, 9202009; Aryogen Pharmed, Teheran, Iran) were used, which will be called Altebrel-6, 8, 9 and Enbrel-G, H1, H4, respectively. Mass spectrometry grade trypsin and rLysC and protein deglycosylation mix from Promega Corporation (Mannheim, Germany), sequencing grade AspN from Roche (Mannheim, Germany), PNGase F from New England Biolabs (Frankfurt, Germany), dimethylsulfoxide (DMSO) and CH_3_I from Sigma-Aldrich Corporation (Darmstadt, Germany), 0.5 mL centrifugal 10 kDa filter and TNF-α from Merck Millipore (Darmstadt, Germany), Alamar blue dye from Invitrogen (Darmstadt, Germany), sensor chip CM5, amine coupling kit and human antibody capture kit from GE Healthcare Life Sciences (Darmstadt, Germany), were used.

### 2.2. Peptides Preparation

Protein digestion was performed on 200 µg of the non-modified or deglycosylated sample diluted in the reducing solution (6 M urea, 100 mM ammonium bicarbonate buffer, 10 mM dithiothreitol) to a final volume of 100 µL. Disulfide bonds were reduced by incubation at 57 °C for 30 min and followed by alkylation with 6 µL of 1 M iodoacetamide and room temperature incubation for 45 min in the dark. After reduction, samples were transferred into a 10 kDa cut-off centrifugal filter and washed 5 times with ammonium bicarbonate buffer at 13,000 rpm. Tryptic and LysC digestion was performed overnight at 37 °C by adding trypsin to the protein solution in a 1:100 ratio (*w*/*w*). The enzyme was then separated from the resulting peptides by centrifugation at 13,000 rpm for 10 min. Tryptic peptides were completely vacuum dried and resuspended in 0.1% formic acid for nano-LC-ESI-MS/MS analysis. Analogously, the digestion by AspN was performed with an enzyme to substrate ratio of 1:20. Deglycosylation using PNGase F or protein deglycosylation mix was performed according to the manufacturer’s instructions. The digested samples were analyzed by nano-LC-ESI-MS/MS using a Dionex UltiMateTM 3000 RSLCnano system (Thermo Fisher Scientific, Bremen, Germany) coupled to an Orbitrap Fusion Tribrid mass spectrometer (Thermo Fisher Scientific, Bremen, Germany). All samples were analyzed in triplicate.

### 2.3. Nano-LC-ESI-MS/MS Peptide Mapping

Mobile phase A was 0.1% formic acid in water and mobile phase B was 0.1% formic acid in acetonitrile. 1 µL (500 ng) of the samples were loaded onto a Thermo Scientific™ Acclaim PepMap™ C18 peptide trapping column (100 μm × 2 cm, 5 μm, 100 Å) at 15 μL/min for 3 min with 2% mobile phase B and separated on reverse phase C18 column (Thermo Scientific™ Acclaim PepMap™ RSLC, 75 μm × 50 cm, 2 μm, 100 Å). After washing the column for 5 min with 3% mobile phase B, the peptides were separated with a linear gradient of 30 min from 3 to 28% of mobile phase B followed by and increased to 35% in 5 min. An increase to 90% of mobile phase B for 9.9 min removed the remaining impurities and the column was re-equilibrated to 3% of mobile phase B for 19.9 min. The column temperature was 45 °C. Electro spray ionization of the eluted peptide was achieved by nanospray Flex™ ion source (Thermo Scientific™) in positive ion mode and the spray was generated at a capillary voltage of 1800 V. Ion transfer tube temperature was set at 300 °C. MS1 scans were acquired (400–1300 *m*/*z*) by Orbitrap mass analyzer at a resolution of 120,000 with a maximum injection time of 120 ms and an AGC Target value of 2 × 10^5^. Most intense peaks were isolated with an isolation window of 1.6 *m*/*z* and an intensity threshold of 1 × 10^4^. After fragmentation by higher energy collision induced dissociation (HCD) at collision energy of 30%, the product ions were detected in the ion trap mass analyzer. MS2 scans were recorded in a data-dependent acquisition mode (DDA) set to top speed mode for precursor ion selection. Dynamic exclusion time was set to 30 s. For ion trap detection, the scan rate was set to rapid, with a fixed first mass (120 *m*/*z*), maximum injection time 60 ms and AGC target value at 1 × 10^4^.

### 2.4. Software Analysis

The data were processed using the Thermo Scientific™ Xcalibur™ 4.0.27.13 software. For primary structure identification, Thermo Scientific™ Proteome Discoverer™ software was mostly used. The amino acid sequence of etanercept was used as the database fasta file. The software parameters were listed as follows: the precursor mass tolerance was 10 ppm; the fragment mass tolerance was 0.2 Da; maximal limitation for 2 missed cleavage of enzyme; minimum peptide length of 6 amino acids, the variable modification was oxidation (M, +15.995 Da), acetyl (Protein N-term, +42.011 Da), amidation (D, E, −0.984 Da) and deamidation (N, Q, +0.984 Da); the fixed modification was carbamidomethyl (C, +57.021 Da); targeted FDR (strict) for PSMs was 1%. However, obtained results from Byonic™ (Protein Metrics Inc., Cupertino, CA, USA.) and MaxQuant (Max Planck Institute of Biochemistry, Munich, Germany) allowed a more complete and precise view of peptide modifications and identification. MaxQuant was mostly used for quantification of methionine oxidation, Byonic™ for glycan site and composition analysis of the glycosylation. For MaxQuant analysis, the software parameters were listed as follows: the peptide tolerance was 20 ppm, MS /MS tolerance was 0.5 Da. maximal limitation for 2 missed cleavage for enzyme; minimum peptide length of 6 amino acids, the variable modification was oxidation (M, +15.995 Da), acetyl (Protein N-term, +42.011 Da) and deamidation (N, +0.984 Da); the fixed modification was carbamidomethyl (C, +57.021 Da). FDR for peptides and protein groups was set 1%. Above parameters were the same among different proteases specificities, such as trypsin, LysC and AspN. For Byonic™ the parameters were listed as follows: the precursor mass tolerance was 3 ppm; the fragment mass tolerance was 0.3 Da; maximal limitation for 2 missed cleavage for enzyme; minimum peptide length of 6 amino acids, the variable modification was oxidation (M, +15.995 Da), acetyl (Protein N-term, +42.011 Da), amidation (D, E, −0.984 Da) and deamidation (N, Q, +0.984 Da); the fixed modification was carbamidomethyl (C, +57.021 Da); the software database for mammalian N/O-glycans was chosen, spectrum-level FDR was set to auto cut and protein FDR cut-off was 1%.

### 2.5. Release and Permethylation of N/O-Glycans

N-glycans were released by PNGase F and further separated from the polypeptides by 10 kDa spin columns. The samples were transferred into a glass tube and dried completely and permethylation reaction was performed based on the classical procedure [19]. Twenty mg of sodium hydroxide particles were dissolved and homogenized in DMSO using a grinder. 200 μL of the homogenized sodium hydroxide was pipetted into the glass tube containing the lyophilized N-glycans. 120 μL of methyl iodide (CH_3_I) was added into each sample and incubated for one hour using a mixer with 550 rpm and room temperature. The reaction was quenched by 200 μL of 5% acetic acid at 4 °C. After mixing for 5 min, 300 μL of chloroform were added to each reaction tube mixed for 5 min and centrifuged at 8000 rcf at 4 °C. The upper (aqueous) phase was discarded and the organic chloroform phase was washed with 400 μL of H_2_O. The aqueous phase was discarded and the washing step was repeated until the aqueous layer reached pH 7. The remaining organic phase, containing permethylated N-glycans, was concentrated and dried. Intact O-glycans were released according to Huang et al. [20]. The dried samples were dissolved in 200 μL of 28% aqueous ammonium hydroxide solution. Then 40 mg of ammonium carbonate powder was added to each reaction mixture and incubated at 60 °C for 40 h using a shaker at 550 rpm. The solution was vacuum dried and repeatedly resuspended in water for removal of ammonium hydroxide and ammonium carbonate. The dried sample was dissolved in 30 µL of 0.5 M boric acid and incubated at 37 °C for 30 min. The boric acid was removed by vacuum drying and repeated resuspension in methanol until salts were no longer visible in the vial. The permethylation procedure was the same with N-glycans.

### 2.6. MALDI-MS and Nano-LC-ESI-MS/MS Analysis of Permethylated Glycans

The permethylated glycans were resuspended in 50% methanol. Each sample was spotted onto a ground steel target and co-crystalized with saturated α-cyano-4-hydroxycinnamic acid (CHCA) dissolved in 50% methanol. MALDI spectra were acquired in positive-reflectron mode ranging *m*/*z* from 500 to 5000. MALDI laser energy was set at 40%. MALDI-MS analysis was done using Ultraflextreme MALDI-MS system (Bruker Daltonik GmbH, Bremen, Germany). For data processing and export of the mass list, flexAnalysis Version 3.3 (Bruker Daltonik GmbH) was used.

Nano-LC-ESI-MS/MS analysis of the permethylated glycans was performed by a DIONEX UltiMate 3000 UHPLC system (Thermo Fisher Scientific) coupled with an Orbitrap Fusion Tribrid mass spectrometer (Thermo Fisher Scientific). The permethylated glycans were dissolved in 0.1% formic acid and separated using nanoC18 column. Mobile phases A and B and the columns are mentioned in the peptide mapping section. The sample was injected onto the trapping column at 2% mobile phase B with the flow rate of 3 µL/min and washed for 10 min. The glycans were eluted onto the analytical column at 10% mobile phase B and separated at a flow rate of 0.2 µL/min with the increase of mobile phase B to 30% in 5 min, to 75% in 70 min and finally to 95% in 80 min. The parameters were set as follows: positive voltage was set at 1.8 kV; the scan range *m*/*z* was 300 to 2000; the collision energy of CID was 35% and MS2 spectra were acquired by DDA (top 20).

### 2.7. Data Analysis for Permethylated Glycans

Since there is no software available for the efficient structural analysis of permethylated glycans, a workflow was designed to process the acquired data. MALDI-MS analysis of the samples provided the intact molecular weight of the permethylated glycan as the first clue for deduction of the permethylated monosaccharide compositions. It was then followed by nano-LC-ESI-MS/MS measurement of the samples. GlycoWorkbench [21] was used to calculate the mass of the MS1 and MS2 ions to be matched with the experimental data from the MALDI-MS and nano-LC-ESI-MS/MS analysis that finally led to the confirmation of the glycan structures. This analytical workflow exemplified with GlcNAc3Man3 is presented in the Supplementary Materials, Figure S1. MALDI-MS analysis of the sample resulted in the detection of sodium adduct GlcNAc3Man3 with the *m*/*z* value of 1416.726. Meanwhile, nano-LC-ESI-MS/MS analysis provided the MS1 and MS2 data that was used and matched manually by the GlycoWorkbench B/C and Y/Z generated ions (from the theoretical precursor ion input). Finally, the N-glycan was characterized and detected confidently.

### 2.8. Functional Assays

Functional assays were performed on one lot of Enbrel^®^ (H17609) and Altebrel™ (9202008). N-deglycosylated samples, negative controls which underwent the same procedure for the N-deglycosylation except adding the PNGase F, and non-modified Enbrel^®^ and Altebrel™ samples were compared directly. Briefly, SPR analysis was carried out at 25 °C using HBS-EP buffer (10 mM HEPES, 150 mM NaCl, 3 mM EDTA and 0.005% polysorbate 20 with pH of 7.4) as running buffer. Amine coupling of the anti-human IgG (Fc) antibody to the CM5 sensor chip surface via carboxyl groups on the dextran was achieved according to the automated immobilization procedure at a density of 2000 RU. Etanercept was captured by the immobilized anti-human Fc with the amount of 100 RU. Five increasing concentrations of TNFα (0.5 to 8 nM) were injected for 180 s. The bound TNFα was allowed to dissociate for 1200 s while washing the chip with running buffer (HBS-EP). The chip surface was regenerated with 4 M MgCl_2_.

The affinity of etanercept to TNF-α was measured via surface plasmon resonance using the capturing method by GE Healthcare Biacore™ X100 (GE Healthcare) in single-cycle kinetics procedure (kinetic titration). All the analyses were carried out in duplicate and the binding kinetic parameters including Ka (association rate constant), Kd (dissociation rate constant) and KD (dissociation equilibrium constant) were fitted to the 1:1 Langmuir interaction model calculated by BIAevaluation software version 2 (GE healthcare).

L929 cells were cultured in 75 cm^2^ flask in DMEM-F12 medium supplemented with 10% FBS. Afterward, 50 µL of the cell suspension (1.6–1.8 × 10^5^ cells/mL) and 50 µL of TNFα (25 ng/mL) were transferred into a 96-well micro plate. Serially diluted Altebrel™ and Enbrel^®^ samples were then added to the different wells and incubated for 72 h at the condition of 37 °C, 5% CO_2_ and 95–98% humidity. After incubation, 30 µL of Alamar blue dye (cell viability indicator) were added and incubated for eight hours. The fluorescence was measured with a FL×800™ fluorescence microplate reader (BioTek, Bad Friedrichshall, Germany) at 540 nm for excitation and 590 nm for emission.

The biological assays were designed based on the necroptotic effect of TNF-α on L929 cells (American Type Culture Collection, Cat No CCL-1). Since the TNF-α is blocked by etanercept, cell death can be neutralized by its presence. The TNF-α blocking power and thus increasing the cell survival rates of a dilution series of etanercept samples were analyzed. Data from the biological assay was analyzed by the PLA 2.0 Bioassay software (Stegmann Systems, Rodgau, Germany) and fitted in a four-parameter logistic curve.

## 3. Results

### 3.1. Analysis of Primary Structure

**Figure 1:**
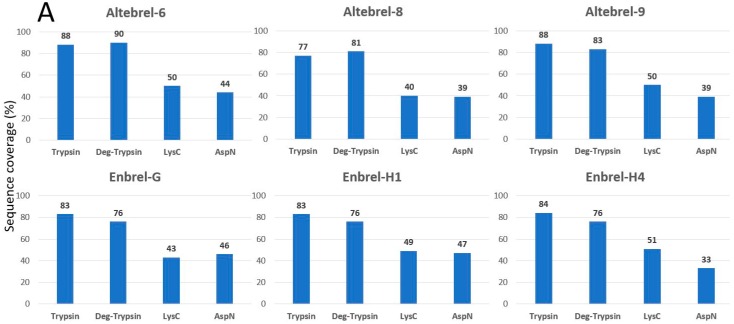

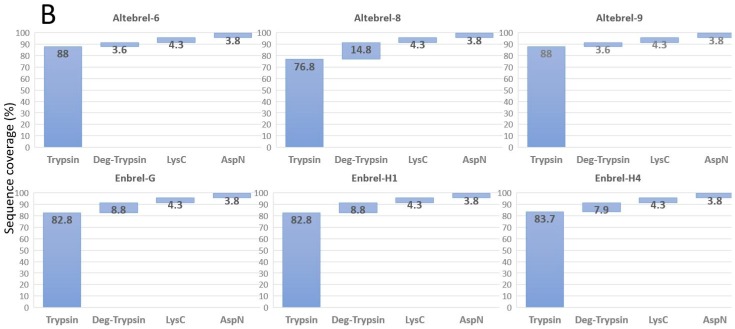
Identified amino acid sequences in percentage after digestion with different proteases and deglycosylation (Deg-trypsin). (**A**) Covered sequence obtained by using each of the proteases or deglycosylation; (**B**) Share of deglycosylation or LysC and AspN in improvement of the covered sequence by trypsin.

To cover the whole primary structure of etanercept, AspN and LysC digestions were performed in parallel to trypsin. Nano-LC-ESI-MS/MS peptide mapping of the different trypsin digested etanercept samples led to the identification of 77% to 88% of the etanercept sequence (Figure 1). N-deglycosylation by PNGase F improved the sequence coverage by 4% to 15% due to the identification of the peptide with an N-glycosylation site at asparagine 317. For identification of the O-glycosylated sites, all three batches of the originator were further deglycosylated by a mixture of O-glycosidase, neuraminidase, β1-4 galactosidase and β-N-acetylglucosaminidase. Consequently, the sequence from serine 202 to lysine 237 from the originator batches was then identified, verifying O-glycosylation in this region. As inferred from Figure 1, not the entire protein sequence can be identified with trypsin due to the lack of cleavable lysine or arginine residues in the range from lysine 120 to proline 158, an important region for the anti-TNF-α property of etanercept. To cover this region, AspN and LysC digestions were performed. This led to the sequence coverage of 99.7%. Since the C-terminal lysine has been cleaved in all batches, a complete sequence coverage could not be achieved. Figure 1 concludes the results for the sequence coverage gained by different proteases and deglycosylation. Moreover, Figure S2 (Supplementary Materials) shows the exact identified parts using the proteases and deglycosylation in Altebrel-6.

### 3.2. Nano-LC-ESI-MS/MS Based Etanercept Peptide Characterization

**Figure 2:**
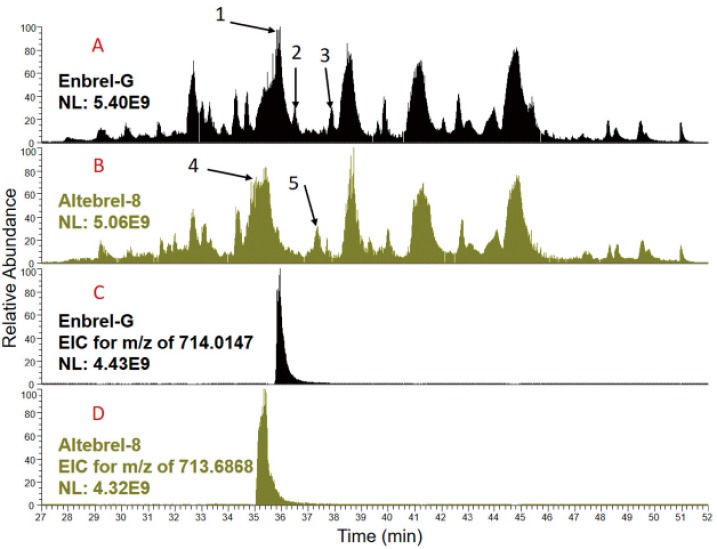
TICs from the originator (**A**) and the proposed biosimilar (**B**) after tryptic digestion. The main differences between the TICs are pointed out with the black arrows. The extracted ion chromatograms (XICs) for the (276)TPEVTCVVVDVSHEDPEVK(294) peptide in Enbrel-G and Altebrel-8 for the non-modified (**C**) and amidated (**D**) peptide at glutamic acid 292 can be observed. The amidated peptide is eluted at a different time compared to its non-modified counterpart.

Peptide mapping of different samples of etanercept were compared using total ion chromatograms (TICs) generated by nano-LC-ESI-MS/MS analysis of trypsin, AspN and LysC (Figure S3 Supplementary Materials). Although generally similar sample profiles were detected, the most obvious differences for the tryptic-digested samples are highlighted in Figure 2 by arrows. Arrows 1, 2 and 3 highlight signals not present in the biosimilar samples and arrows 4 and 5 highlight signals not present in the originator samples. Inspection of the full MS1 spectra of this region around arrow 1 for Enbrel-G and arrow 4 for the Altebrel-8 (Supplementary Materials, Figure S4 parts A and B) indicates triply and doubly charged ions present in both which differed by 0.984 Da ([M + 3H]3^+^: 714.0148 vs. 713.6868 and [M + 2H]2^+^: 1070.5183 vs. 1070.0262). A retention time shift (Figure 2) together with a mass shift of 0.984 Da implies a deamidation. Investigation of the identified peptides by the software analysis (Thermo Scientific Proteome Discoverer™) and MS2 spectra collected for these ions, led to the recognition of the peptide with the sequence of (276)TPEVTCVVVDVSHE DPEVK(294), as the source of TICs differences. This data showed that within the Altebrel™ samples, the amidated peptide (arrow 1) was more intense than the canonical peptide sequence, whereas the canonical peptide (arrow 4) was the most intense peptide in the originator samples.

As indicated by Figure 2, similar results were observed for arrows 2 and 3 in Enbrel-G and arrow 5 in Altebrel-8, in which the peptide (295)FNWYVDGVEVHNAK(308) is responsible for the differences in the TICs. Variants of this peptide were identified with either an amidation at glutamic acid 303 around arrow 2 or a deamidation at asparagine 306 around arrow 3 (Supplementary Materials, Figure S4 parts C and D).

Besides amidation and deamidation, other commonly occurring amino acid modifications were also analyzed (Supplementary Materials Table S1). In Table 1, methionine oxidation rates were compared by the signal intensity of the oxidized versus the non-oxidized methionine containing peptides. Interestingly, a large variance in oxidation rates on different methionine residues were observed (Table 1). The peptide (437)WQQGNVFSCSVMHEALHNHYTQK(459), had the lowest oxidation rate, whereas (269)DTLMISR(275), with almost 60%, showed the highest methionine oxidation rate at Met 272.

### 3.3. Identification of Glycosylation Sites

Byonic™ software was used to interpret MS/MS spectra generated from tryptic digests of etanercept fragmented by HCD to identify glycosylated etanercept peptides. All three proposed N-glycosylation sites were identified, including the asparagine 149 and 171 in the TNFr domain and asparagine 317 in the Fc domain of etanercept. The N-glycosylated peptides can be found in Table 2 and were conserved across Altebrel™ and Enbrel^®^ batches. The diversity of attached N-glycans to the sites in the TNFr was quite large, with many suggested compositions. For the N-glycosylated peptide in Fc domain, only a few possible glycan compositions were suggested by the software.

In total, five O-glycosylated peptides were identified by the Bionic™ software (Table 2). O1 was located at the N-terminal site of etanercept. O2, O3 and O4 were located at the linker domain in the C-terminal site of the TNFr domain and O5 was found at the hinge region of the etanercept Fc domain. Glycopeptide O2 had seven possible glycosylation sites, one N-glycosylation at asparagine 171 and six possible O-glycosylations where the site localization could not be identified. O3 possesses three probable sites and the O4 glycopeptide contains in total 11 serine/threonine and nine proline units that can be an indication of high O-glycosylation levels. The glycopeptide O4 was identified in the Altebrel™ samples digested with trypsin but not the originator samples.

Analysis of MS/MS spectra from the tryptic digested biosimilar samples confirmed high levels of O-glycosylation at seven possible threonine and serine sites of the glycopeptide. Matched oxonium ions and peptide b and y fragment ions also confirmed the O-glycosylation of glycopeptide O5 at Serine 259 (Supplementary Materials Figure S5) located at the beginning of the Fc subunit.

### 3.4. MALDI-MS Analysis of Permethylated N-Glycans

MALDI-MS analysis was performed after the release of N-glycans with PNGase F and permethylation. Using Enbrel-G, 27 different structures of N-glycan species were identified, mostly with sodium or potassium adducts (Supplementary Materials Figure S6), which were all confirmed by MS1 and MS2 spectra from the nano-LC-ESI-MS/MS analysis to match the identified N-glycosylation sites.

### 3.5. Nano-LC-ESI-MS/MS Analysis of Permethylated Glycans

Nano-LC-ESI-MS/MS was performed to further analyze the glycans. The BPCs (Base peak chromatograms) of the six permethylated glycan samples from the Altebrel™ and Enbrel^®^ (Supplementary Materials Figure S7) showed some minor differences. However, in general, they were similar in terms of retention time and peak intensities. The XICs of several N-glycans from Enbrel-G stated that all of them had isomeric structures with distinct retention times, which resulted from anomers (Supplementary Materials Figure S8). XIC of the N-glycan with the precursor mass of 2017.0369 Da resulted in three distinguishable peaks (Supplementary Materials Figure S8B). The MS2 spectra were obtained for those peaks with the same precursor mass and the fragmentation pattern was quite similar except for the relative intensities of fragment ion at 1566.9272 (Supplementary Materials Figure S8C).

**Figure 3:**
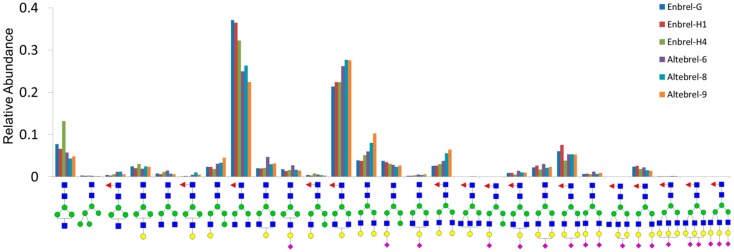
Relative quantities of the N-glycans according to area under the curve of their XICs.

In addition, all N-glycans were relatively quantified using the ratio of the area under the curve for each N-glycan structure in the six different samples (Figure 3). Relative concentrations of the different N-glycan structures across all samples were similar. However, t-test analysis revealed significant differences between the relative abundance for some N-glycans. For example, the GlcNAc4Man3Fuc1 (G0F) and GlcNAc4Man3Fuc1Gal1 (G1F) were the two most abundant N-glycans of etanercept in all the samples. However, G1F was more abundant in the originator samples while G0F had the highest quantity in the proposed biosimilar samples. Accordingly, GlcNAc5Man3Gal2NeuAc1Fuc1, Glc-NAc5Man3Gal3NeuAc2Fuc1 and GlcNAc5Man3Gal3NeuAc3 Fuc1 were the least abundant glycans detected, which were also identified clearly in MALDI-MS.

For O-glycan analysis, glycans were detached from etanercept using β-elimination followed by permethylation, prior to MALDI-MS and nano-LC-ESI-MS/MS. This led to the identification of three types of O-glycan species (Table 3).

Furthermore, numerous new structures were also spotted in this study. These newly found structures are mainly low abundant N-glycans. They were identified in this study because of the sensitivity increase after permethylation. All the newly identified and previously reported N-glycans were summarized in Supplementary Materials Table S2.

Comparison of the BPCs of different samples (Supplementary Materials Figure S9) also showed high similarity between the samples, which possess the same O-glycan composition. For the O-glycan GalNAc1Gal1NeuAc1, four isomeric structures were extracted (Supplementary Materials Figure S10A, peaks a, b, c and d) and the MS2 spectra collected at different retention times were compared (Supplementary Materials Figure S10B). The fragment ions confirmed the linear structure of the O-glycan and the different chromatographic behavior of this structure resulted from glycosidic bond types or binding site.

### 3.6. Functional Assays

To identify the effect of N-deglycosylation on the functionality of etanercept, SPR and cell-based necroptosis neutralization assays were performed. The experiments were conducted using straight from the stock (non-modified), N-deglycosylated and treatment control etanercept, where it was incubated overnight at 37 °C in the PNGase F digestion buffer without the enzyme. This will ensure that the other experimental variables besides N-deglycosylation had not changed the function of etanercept. All the samples were compared in parallel.

KD values of different samples gained by the single-cycle kinetics method using a Biacore^®^ instrument (Supplementary Materials Figure S11) are listed in Table 4. The KD values obtained for the Enbrel were not statistically different (Student’s *t*-test, *p* = 0.05). Also, KD values of etanercept versus the treatment control and the N-deglycosylated forms were not significantly different.

The potency of etanercept was assessed based on the fact that TNF-α induces necroptosis in L929 murine fibroblast cell line model [22]. The potency ratio of the Enbrel^®^ and Altebrel™ samples in N-glycosylated and N-deglycosylated forms compared to the pool of three batches of the originator as the reference (Table 5), demonstrated similar potencies. Likewise, comparing N-glycosylated to N-deglycosylated etanercept did not show significant differences.

## 4. Discussion

With the patent terms of several best-selling biopharmaceuticals nearing their end, introduction of suitable alternatives and developing reliable approaches to demonstrate biosimilarity of these novel substitutes are essential. In the present study, primary structure and some of the post-translational modifications of Altebrel™ were compared side-by-side to those of Enbrel^®^, as the reference drug.

Our results of primary structure analysis are in accordance with the findings of Cho et al. [7] for Benepali^®^, who reported 100% sequence coverage of etanercept using trypsin, LysC and AspN (except that the C-terminal lysine was not identified in the present study). Another comparability study between the originator and the biosimilar (Infinitam^®^) showed 97.9% coverage of the primary sequence, which is surprisingly high, using trypsin as the only protease [23]. Furthermore, the publications of Hofmann et al. [24] and Maity et al. [25] respectively on Erelzi^®^ and Avent™, contain no data about the sequence coverage of the biosimilar etanercept with respect to the amino acid sequence. In none of these comparability studies TICs are shown and compared deeply and this study is the first one which tried to decipher the differences between the TICs.

Peptide mapping using staged TICs of the originator and the proposed biosimilar demonstrated similar peak intensity, comparable elution profile and peptide retention times (Figure S3 Supplementary Materials). However, differences were also observed in some areas. In both Enbrel-G and Altebrel-8, the peptide was identified with and without amidation at glutamic acid 292. The results showed that the degree of amidation in the Altebrel™ samples was much higher than the originator. In other words, higher population of the etanercept proteoform with amidation at glutamic acid 292 was found in comparison with the originator samples.

The oxidation of methionine is an important indicator for oxidative stress conditions during production. Methionine oxidation can occur either during bioprocessing or through analytical sample preparation. Oxidation is one of the major stresses that affect stability and methionine oxidation might result in defects in the structure, safety, potency and efficacy of etanercept. Therefore, the degree of methionine oxidation was investigated and statistical analysis (Student’s *t*-test, *p* = 0.05) showed no differences in oxidation rates between the Enbrel^®^ and Altebrel™. However, since the methionine oxidation rate across different batches of etanercept remained similar, it can be assumed that all the samples were under similar oxidative stress conditions. A study from Cho et al. [7] reported different oxidation levels for two sensitive methionine residues of the biosimilar Benepali^®^ compared with EU sourced Enbrel^®^, which were most likely caused by different environmental, sample preparation and storage conditions or different sources of the samples.

Despite the fact of high oxidation rates on some methionine residues, a recent study found that oxidation has no influence on the bioactivity of etanercept [15]. However, tracking the methionine oxidation is of importance in comparability studies since it can serve as the protectant against oxidative stress [16] and can protect the protein from degradation. Our data implied that methionine 272 which was the most sensitive methionine for oxidation is located in the CH2 domain and indicate that this region is highly prone to modifications such as amidation, deamidation and oxidation. It should be noted that, data about methionine oxidation in this study are not reliable concerning the original state of oxidation, since all experiments were not performed in the absence of oxygen.

Due to the heterogeneous nature, the mass spectrometric characterization of etanercept is challenging. In this study, nano-LC-ESI-MS/MS analysis further showed that all the samples were truncated at the C-terminal (initial leucine can be removed) and the N-terminal end (terminating lysine was removed). As previously stated, C-terminal lysine loss has no significant effect on the antibody structure, thermal stability, antigen binding and potency, FcRn binding and pharmacokinetics in rats [26]. Some differences in deamidation between the biosimilar and the originator were observed. However, the observed differences are due to the modifications, not mutations or other structural deficiencies. Kwon et al. have listed the critical attributes of etanercept and deamidation was not considered to have a big impact on the functionality of etanercept and might not decrease the functionality of this molecule [27]. Therefore, the differences in levels of amidation or deamidation observed in this study between the proposed biosimilar and the originator peptides may not affect the functionality of etanercept. In total, it was shown that the primary sequence in all the samples were the same which is a critical quality attribute.

Glycosylation analysis signified that N-glycosylated peptides were conserved in all the batches. O-glycopeptide analysis is more challenging because of more diverse core structures as well as the problem of the site specificity when multiple serine and threonine are present. Proline at +3 and/or -1 can guide to identify the correct O-glycosylation site. Further, biologically closely spaced glycosylated serine/threonine are preferred, whereas aromatic amino acids, cysteine and amino acids with bulky side chains are unfavorable to O-glycosylation [28]. Interestingly, serine, threonine and proline have high occurrence in the linker domain, which might be the rationale of the high levels of O-glycosylation in this area. The obtained results demonstrated the same glycosylation sites in both Enbrel^®^ and Altebrel™ samples. To identify the N/O-glycan composition, derivatization or dependent analysis of the N/O-glycans should be performed.

MALDI-MS is a powerful tool for glycan analysis due to high sensitivity and generating singly charged ions. Glycan chains carrying sialic acid are labile and commonly fragmented during ionization. This can be prevented by permethylation [29]. All permethylated N-glycans were matched with the corresponding structures to the three N-glycosylation sites. Many identified structures had been published before [8]. However, using permethylation increased the sensitivity toward low abundancy N-glycans allowing numerous new structures to be identified in this study.

Despite the advantages of MALDI-MS for the analysis of permethylated glycans, the isomers could not be distinguished by this technique. After permethylation of the glycans, the hydrogens of hydrophilic chemical groups, such as hydroxyl, carboxyl and imino, were displaced by the methyl group. These methyl groups increase the hydrophobicity of the glycans so they can be retained on a C18 column. Therefore, nano-LC-ESI-MS/MS was employed to further analyze the glycan profile of the etanercept. Herein, all the newly identified and previously reported N-glycans (summarized in Supplementary Materials Table S2) were confirmed with higher mass accuracy than the previous report [8], which indicated another advantage of the permethylation strategy for glycan analysis. Permethylation of N-glycans increased the ionization efficiency, which was advantageous to get information from the glycans with the low abundancy and high molecular mass. The combination of MALDI-MS and nano-LC-ESI-MS/MS provide more molecular and structural information than each of them alone. This approach enables us to identify and quantify the glycan structures in detail, which cannot be maintained by HPLC glycan mapping and similar methods.

Surprisingly, randomized control trials on some biosimilars such as Davictrel, Benepali and Erelzi showed that the levels of anti-drug-antibodies production were less than with Enbrel^®^ [30]. Therefore, the biosimilars could lead to the better medication than the originators in some cases. However, the functionality and immunogenicity of the biosimilars should be carefully analyzed since no risk can be tolerated when it is about the human life. For checking the functionality, SPR was used to understand the kinetics of association (Ka), dissociation (Kd) and consequently, affinity (KD) of the etanercept to TNF-α [31]. Analysis of KD values of the proposed biosimilar, reference etanercept and the control samples indicated that N-deglycosylation had no considerable effect on the affinity of etanercept to TNF-α. It also demonstrated that Enbrel^®^ and Altebrel™ bind TNF-α with a similar affinity.

The potencies of Enbrel^®^ and Altebrel™ to induce necroptosis proved to be comparable. As a TNF inhibitor drug, etanercept neutralizes TNF-α with increasing concentrations and therefore led to a higher degree of cell survival [7,32]. The exact effect of etanercept affinity on the in-vitro cell survivals is not fully understood; however, the findings here were in accordance with the affinity values from the SPR analysis and further confirms that N-glycosylation in etanercept had no significant effect on the in-vitro biological activity and affinity of etanercept to its target. To date, it is the first report that connects the N-glycosylation to the in-vitro functionality of etanercept. Still, the impact of N-glycosylation on the immunogenicity and in-vivo functionality needs to be investigated. It is clear that glycosylation is one of the main characteristics of a molecule, but the importance of glycosylation as a critical quality attribute should be defined case by case and differs in different biotherapeutics.

Mass spectrometry characterization of Enbrel^®^ and Altebrel™ proved the similarity of their amino acid sequences. However, minor differences in the TICs have been observed and comprehensive analysis of the TICs led to the identification of the molecular basis for the inequalities between Enbrel^®^ and Altebrel™, which was related to the different degrees of peptide amidation and deamidation. These findings would enable the manufacturers to find and avoid the sources of deviations. Furthermore, applying permethylation derivatization technique contributed to the better identification and relative quantification of the N-glycans and three O-glycan structures in etanercept were identified. Recently, the monograph of etanercept was published in the Supplement 9.5 of the European Pharmacopoeia and became effective on 1 July 2018 [33]. In the monograph, HPLC glycan mapping has been advised to be used. This method is separating N-glycans into nine peaks and the sum of the areas of the peaks due to the neutral and sialylated N-glycans should be evaluated. However, no ranges have been suggested and it was entrusted to the competent authority. Consequently, in similar publications just the qualitative comparison has been made to make sure that no unique peak would be detected [7]. Our study has the thorough data on the complete identification and quantification of the N-glycan, which are comparable in Altebrel™ and Enbrel^®^. Moreover, functional assays were performed on N-deglycosylated samples to understand the impact of N-glycosylation on in-vitro properties of etanercept. In this case, N-glycosylation has no significant impact on in-vitro functions of etanercept and all the samples showed similar activity. It is clear that the further analysis representing the biological, functional and clinical characteristics of etanercept is needed to examine the exact relations between the functional properties and glycosylation.

Analysis of several reference drug batches over time was the basis for defining the acceptable ranges of variations in the product or the biosimilarity goal posts. These findings which analyzed the activity in relation to the structure can be used for determination of the biosimilarity goal posts and etanercept critical quality attributes. To further define the biosimilarity goal posts, in vivo experiments and analysis of the samples with the accelerated modifications such as methionine oxidation can be examined. In conclusion, the present study describes a detailed strategy for the mass spectrometric characterization of etanercept primary sequence and glycosylation as a model and investigates the effect of N-glycosylation on its in-vitro functional properties. It also can serve the regulatory board to establish the requirements needed for biosimilarity or interchangeability demonstration by providing the further information about the glycosylation, primary structure and functionality of etanercept. Moreover, the same primary structure, comparable N-glycan profile, comparable rates of methionine oxidation and in-vitro functionality (based on Student’s t-test analysis), stated a high similarity of Altebrel™ to Enbrel^®^ in this regard.

## Figures and Tables

**Table 1:** Oxidation levels at various methionine sites in percentage.

Met Position	Altebrel-6	Altebrel-8	Altebrel-9	Enbrel-G	Enbrel-H1	Enbrel-H4
30	31	29	32	31	36	28
187	42	22	36	51	30	50
272	59	58	60	58	56	61
378	35	33	28	35	31	32
448	4	4	3	4	3	3

**Table 2:** Confirmed N/O-glycan sites of the tryptic glycopeptides.

O-glycopeptide	O1	LPAQVAFTPYAPEPGSTCR
	O2N2	PHQICNVVAIPGNASMDAVCTSTSPTR
	O3	SMAPGAVHLPQPVSTR
	O4	SQHTQPTPEPSTAPSTSFLLPMGPSPPAEGSTGDEPK
	O5	SCDKTHTCPPCPAPELLGGPSVFLFPPKPK
N-glycopeptides	N1	CRPGFGVARPGTETSDVVCKPCAPGTFSNTTSSTDICR
	O2N2	PHQICNVVAIPGNASMDAVCTSTSPTR
	N3	EEQYNSTY

**Table 3:** The list of identified O-glycans.

*O*-Glycan Structures	Natural Monoisotopic Mw	Permethylation Monoisotopic Mw		Deviation (ppm)	Byonic Software	
		Experiment	Theory		Peptide	Score
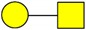	383.1428	495.2674	495.2680	−1.21147	K.THTC[+57.021]PPC[+57.021]PAPELLGGP**S**[+365.132]VFLFPPKPK.D	643.8
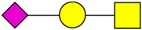	674.2382	856.4411	856.4416	−0.58381	K.THTC[+57.021]PPC[+57.021]PAPELLGGP**S**[+656.228]VFLFPPKPK.D	831.9
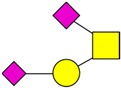	965.3336	1217.6147	1217.6153	−0.49277	K.THTC[+57.021]PPC[+57.021]PAPELLGGP**S**[+947.323]VFLFPPKPK.D	950.7

**Table 4:** KD values of the etanercept samples in pM.

Samples	KD-Replicate1 (pM)	KD-Replicate 2 (pM)	KD Average (pM)
N-deg Altebrel-8	14.5	19.9	17.2
Control Altebrel-8	12.0	17.1	14.5
Altebrel-8	14.7	11.5	13.1
N-deg Enbrel H1	10.2	19.8	15.0
Control Enbrel-H1	15.3	14.7	15.0
Enbrel H1	11.6	10.2	10.9

**Table 5:** Potency ratio of the etanercept samples to the in-house standard.

Samples	Replicate 1	Replicate 2	Average
N-deg Altebrel-8	0.79	0.95	0.87
Control Altebrel-8	0.84	0.91	0.88
N-deg Enbrel H1	0.79	0.95	0.87
Control Enbrel-H1	0.85	1.08	0.97

## Supplementary Materials

The following are available online at http://www.mdpi.com/1424-8247/12/1/14/s1, Figure S1: Workflow for the analysis of glycan structures via permethylation. Figure S2: Sequence of etanercept with the identified peptides after peptide mapping using the tryptic peptide, deglycosylated tryptic peptide (Deg), LysC and AspN digested peptides in Altebrel-6 is compared. Figure S3: TICs of different etanercept digested by A) trypsin B) AspN and C) LysC. Figure S4: Full MS1 and MS2 spectra of the (276)TPEVTCVVVDVSHEDPEVK(294) and (295)FNWYVDGVEVHNAK(308) peptide in Enbrel-G and Altebrel-8. Figure S5: Fragment spectrum of the glycopeptide containing the O-glycosylation site at O5 peptide with following the oxonium fragment ions (labeled by the full name) at MS2 level. Figure S6: MALDI-MS mass spectrum of permethylated N-glycans released from Enbrel-G. Figure S7: BPCs of permethylated N-glycans derived from different etanercept samples from two vendors. Figure S8: Chromatograms and spectra from N-glycans from Enbrel-G representing different isomers. Figure S9: BPCs of O-glycans from different vendors. Figure S10: Chromatograms and spectra of O-glycans from Enbrel-G showing different isomers. Figure S11: Single cycle kinetics graph of Altebrel-8 with the fitted model. Figure S12: Dose-response curves of (A) Control Enbrel-H1 and (B) Control Altebrel-8 versus the in-house reference standard. Table S1: List of observed modifications in etanercept. Table S2: Identified N-glycans in Enbrel-G.
