# Supplementary material for: Structural and In Vitro Functional Comparability Analysis of Altebrel™, a Proposed Etanercept Biosimilar: Focus on Primary Sequence and Glycosylation
Source: Pharmaceuticals (Basel). 2019 Jan 17;12(1):14. doi: 10.3390/ph12010014 (PMC6469174; doi:10.3390/ph12010014)
Supplement: Supplementary file 1 [file pharmaceuticals-12-00014-s001.pdf]

Supporting Information

Structural and In Vitro Functional Comparability Analysis of Altebrel™, a Proposed Etanercept Biosimilar: Focus on Primary Sequence and Glycosylation

Ramin Fazel [1,2,†], Yudong Guan [2,†], Behrouz Vaziri [3], Christoph Krisp [2], Laura Heikaus [2], Amirhossein Saadati [4], Siti Nurul Hidayah [2], Manasi Gaikwad [2] and Hartmut Schlüter [2,*]

[1] Department of Biotechnology, College of Science, The University of Tehran, 1417864311 Tehran, Iran; Ramin.Fazel@gmail.com. Ramin.Fazel@ut.ac.ir

[2] Mass Spectrometric Proteomics, Institute of Clinical Chemistry and Laboratory Medicine, Campus Forschung, N27 Raum 00.008, Universitätsklinikum Hamburg-Eppendorf, Martinistr. 52, 20246 Hamburg, Germany; y.guan@uke.de (Y.G.); c.krisp@uke.de (C.K.); l.heikaus@uke.de (L.H.); s.hidayah@uke.de (S.N.H.); m.gaikwad@uke.de (M.G.)

[3] Biotechnology Research Center, Pasteur Institute of Iran, 1316943551 Tehran, Iran; Behrouz-vaziri@pasteur.ac.ir

[4] AryoGen Pharmed, Cross Tajbakhsh Street, 24th Kilometer Makhsous, Tehran, Iran; saadatirada@aryogen.com

* Correspondence: hschluet@uke.de; Tel.: +49(0)40-7410-58795

† These authors contributed equally to this work.

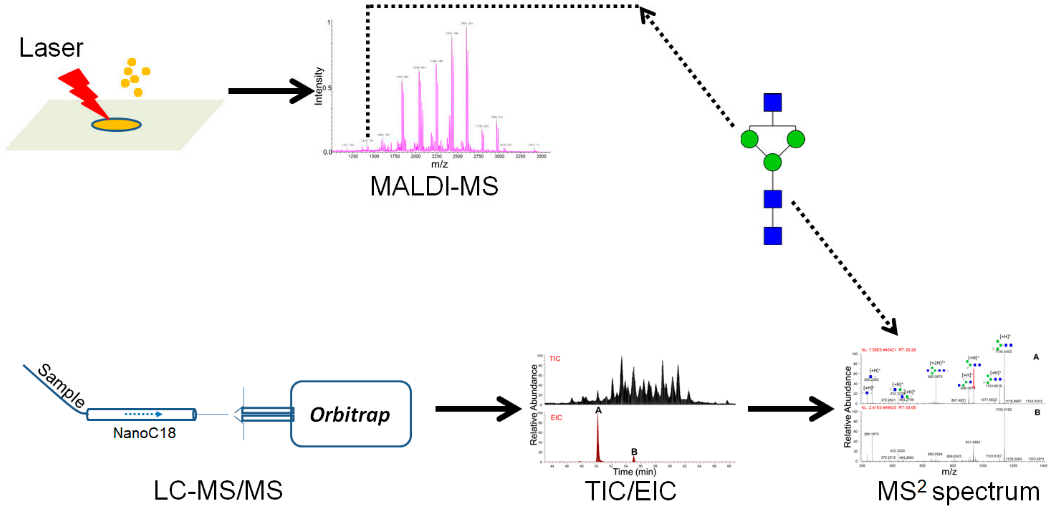

Figure S1: Workflow for the analysis of glycan structures via permethylation

Figure S2: Sequence of etanercept with the identified peptides after peptide mapping using the tryptic peptide, deglycosylated tryptic peptide (Deg), LysC and AspN digested peptides in Altebrel-6 is compared. The blank areas show the unidentified peptides by each of the methods. Aspartic acid 235 is the boundary of the TNFr and Fc domains.

		1	11	21	31	41	51	61	71	81	91
1	**Sequence**	LPAQVAFTPY	APEPGSTCRL	REYYDQTAQM	CCSKCSPGQH	AKVFCTKTSD	TVCDSCEDST	YTQLWNWVPE	CLSCGSRCSS	DQVETQACTR	EQNRICTCRP
	**Trypsin**	LPAQVAFTPY	APEPGSTCRL	REYYDQTAQM	CCSKCSPGQH	AKVFCTKTSD	TVCDSCEDST	YTQLWNWVPE	CLSCGSRCSS	DQVETQACTR	ICTCRP
	**Deg+Try**	LPAQVAFTPY	APEPGSTCRL	REYYDQTAQM	CCSKCSPGQH	AKVFCTKTSD	TVCDSCEDST	YTQLWNWVPE	CLSCGSRCSS	DQVETQACTR	EQNRICTCRP
	**LysC**	LPAQVAFTPY	APEPGSTCRL	REYYDQTAQM	CCSKCSPGQH	AK					
	**AspN**	LPAQVAFTPY	APEPGSTCRL	REYYDQTAQM	CCSKCSPGQH	AKVFCTKTS	DST	YTQLWNWVPE	CLSCGSRCSS		
101	**Sequence**	GWYCALSKQE	GCRLCAPLRK	CRPGFGVARP	GTETSDVVCK	PCAPGTFSNT	TSSTDICRPH	QICNVVAIPG	NASMDAVCTS	TSPTRSMAPG	AVHLPQPVST
	**Trypsin**	GWYCALSKQE	GCRLCAPLRK				PH	QICNVVAIPG	NASMDAVCTS	TSPTRSMAPG	AVHLPQPVST
	**Deg+Try**	GWYCALSKQE	GCRLCAPLRK				PH	QICNVVAIPG	NASMDAVCTS	TSPTRSMAPG	AVHLPQPVST
	**LysC**			CRPGFGVARP	GTETSDVVCK						
	**AspN**				DVVCK	PCAPGTFSNT	TSSTDICRPH	QICNVVAIPG	NASM		
201	**Sequence**	RSQHTQPTPE	PSTAPSTSFL	LPMGPSPPAE	GSTGDEPKSC	DKTHTCPPCP	APELLGGPSV	FLFPPKPKDT	LMISRTPEVT	CVVVDVSHED	PEVKFNWYVD
	**Trypsin**	RSQHTQPTPE	PSTAPSTSFL	LPMGPSPPAE	GSTGDEPKSC	DKTHTCPPCP	APELLGGPSV	FLFPPKPKDT	LMISRTPEVT	CVVVDVSHED	PEVKFNWYVD
	**Deg+Try**	RSQHTQPTPE	PSTAPSTSFL	LPMGPSPPAE	GSTGDEPKSC	DKTHTCPPCP	APELLGGPSV	FLFPPKPKDT	LMISRTPEVT	CVVVDVSHED	PEVKFNWYVD
	**LysC**				SC	DKTHTCPPCP	APELLGGPSV	FLFPPKPKDT	LMISRTPEVT	CVVVDVSHED	PEVKFNWYVD
	**AspN**				DEPKSC	DKTHTCPPCP	APELLGGPSV	FLFPPKPKDT	LMISRTPEVT	CVVVDVSHED	PEVKFNWYV
301	**Sequence**	GVEVHNAKTK	PREEQYNSTY	RVVSVLTVLH	QDWLNGKEYK	CKVSNKALPA	PIEKTISKAK	GQPREPQVYT	LPPSREEMTK	NQVSLTCLVK	GFYPSDIAVE
	**Trypsin**	GVEVHNAKTK	PR	VVSVLTVLH	QDWLNGKEYK	CKVSNKALPA	PIEKTISKAK	GQPREPQVYT	LPPSREEMTK	NQVSLTCLVK	GFYPSDIAVE
	**Deg+Try**	GVEVHNAKTK	PREEQYNSTY	RVVSVLTVLH	QDWLNGK	CKVSNKALPA	PIEKTISKAK	GQPREPQVYT	LPPSREEMTK	NQVSLTCLVK	GFYPSDIAVE
	**LysC**	GVEVHNAK				ALPA	PIEK	GQPREPQVYT	LPPSREEMTK	NQVSLTCLVK	GFYPSDIAVE
	**AspN**										DIAVE
401	**Sequence**	WESNGQPENN	YKTTPPVLDS	DGSFFLYSKL	TVDKSRWQQG	NVFSCSVMHE	ALHNHYTQKS	LSLSPGK			
	**Trypsin**	WESNGQPENN	YKTTPPVLDS	DGSFFLYSKL	TVDKSRWQQG	NVFSCSVMHE	ALHNHYTQK	LSLSPG			
	**Deg+Try**	WESNGQPENN	YKTTPPVLDS	DGSFFLYSKL	TVDK WQQG	NVFSCSVMHE	ALHNHYTQK	LSLSPG			
	**LysC**	WESNGQPENN	YKTTPPVLDS	DGSFFLYSK	SRWQQG	NVFSCSVMHE	ALHNHYTQK				
	**AspN**	WESNGQPENN	YKTTPPVLDS	DGSFFLYSKL	TV						

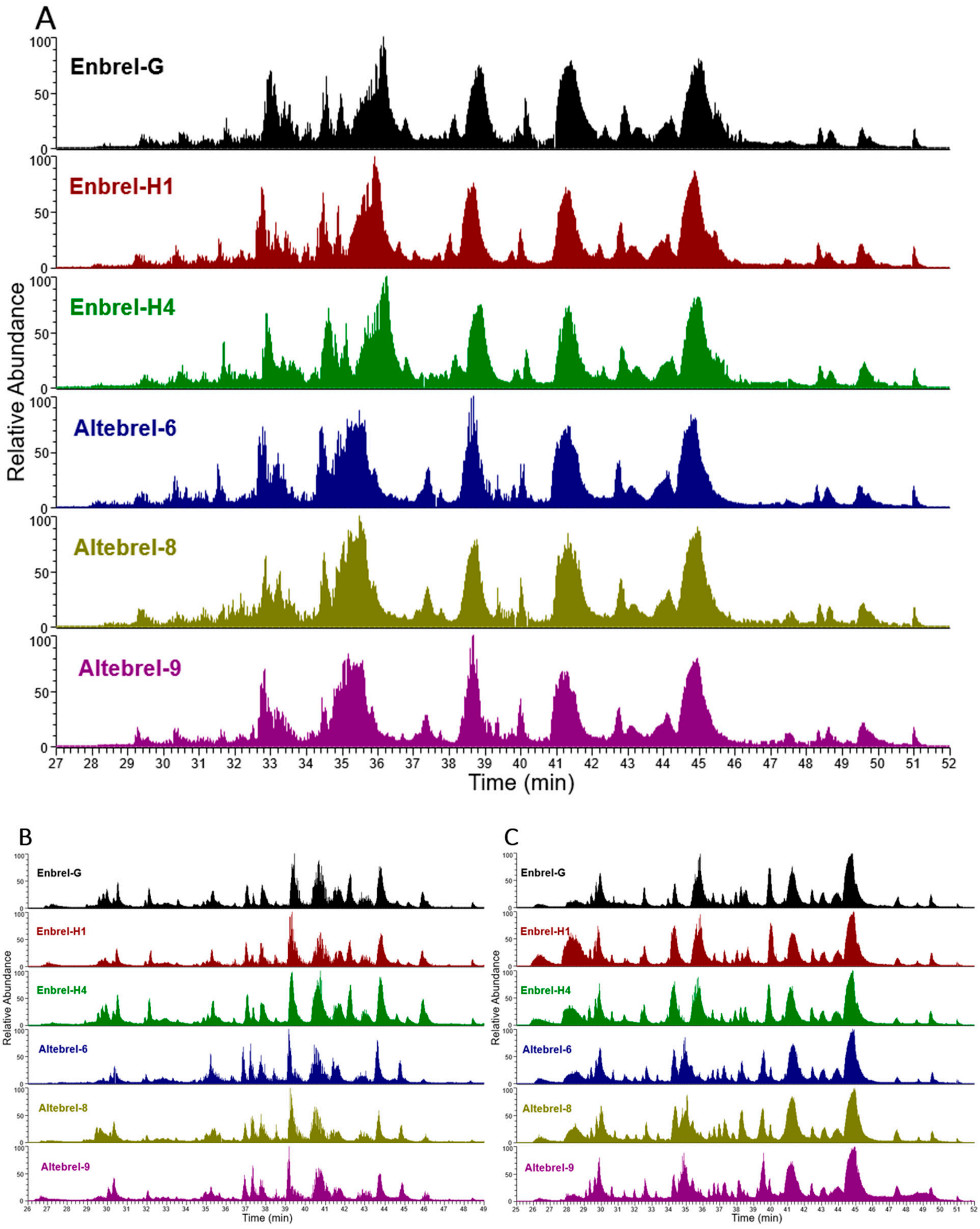

Figure S3: TICs of different etanercept digested by A) trypsin B) AspN and C) LysC.

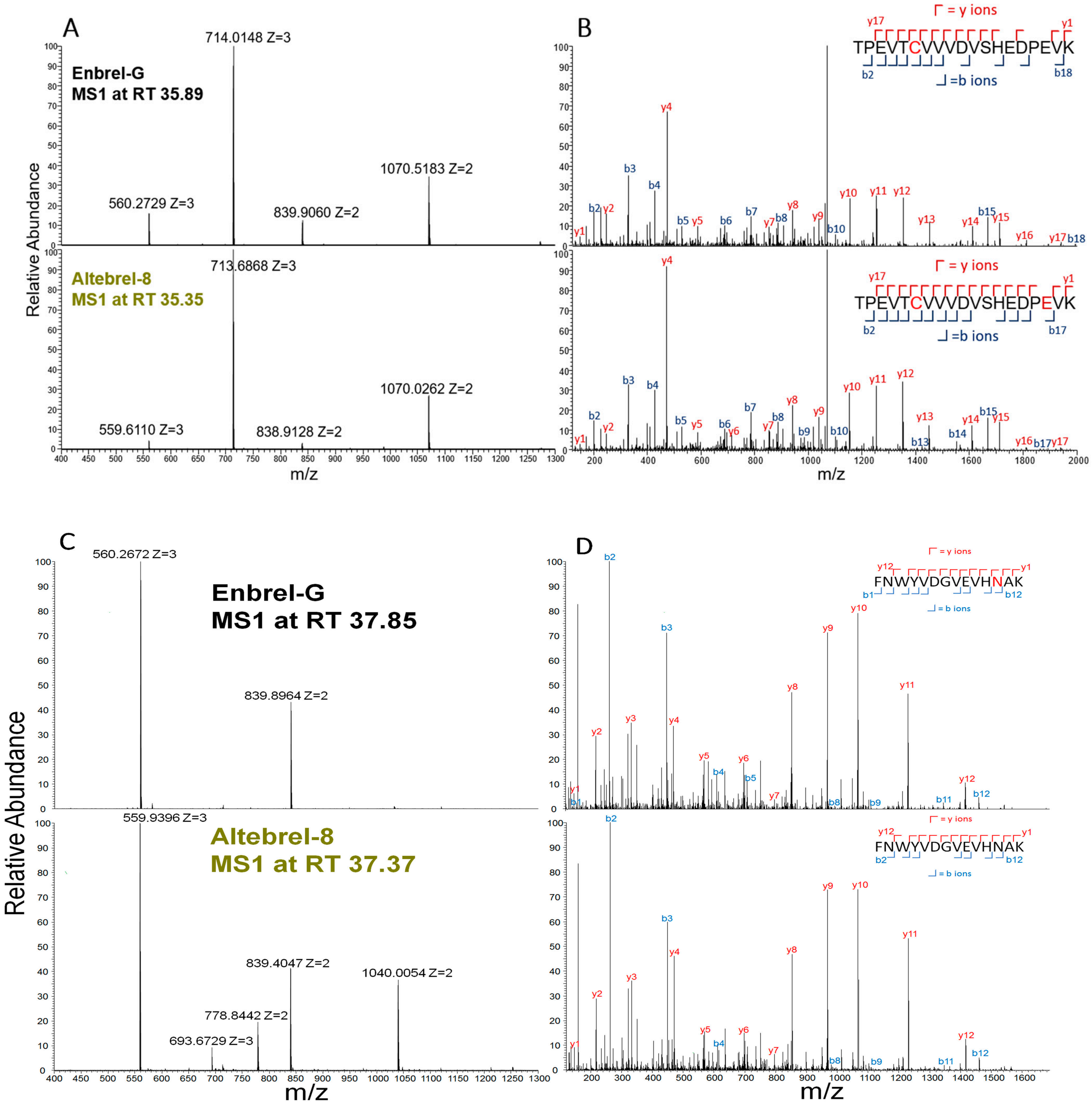

Figure S4: A) Full MS1 spectra of the (276)TPEVTCVVVDVSHEDPEVK(294) peptide in Enbrel-G and Altebrel-8. The peaks with the *m/z* values of 714.0148 and 1070.5183 are triply and doubly charged states of the precursor ion in Enbrel-G whereas the peaks with the values of 713.6868 and 1070.0262 are the ones in Altebrel-8. B) MS2 fragment spectra of the non-modified and amidated peptide at glutamic acid 292 with the assigned fragments. Because of the reduction and alkylation, the cysteine 281 is carbamidomethylated in both samples. C) Full MS1 spectra of the (295)FNWYVDGVEVHNAK(308) peptide in Enbrel-G and Altebrel-8. The peaks with the m/z of 560.2672 and 839.8964 are triply and doubly charged states of the precursor ion in Enbrel-G whereas the peaks with the values of 559.9396 and 839.4047 are the ones in Altebrel-8. D) MS2 fragment spectra of the non-modified and deamidated peptide at Asparagine 306 with the assigned fragments.

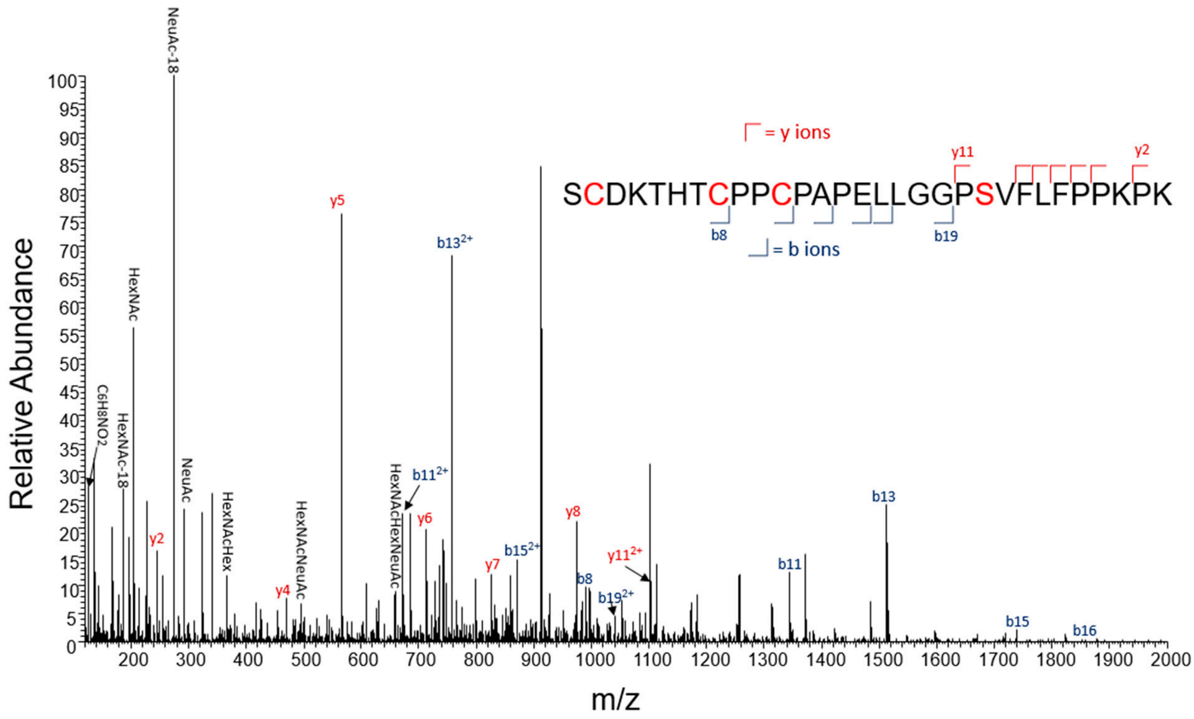

Figure S5: Fragment spectrum of the glycopeptide containing the O-glycosylation site at O5 peptide with following the oxonium fragment ions (labeled by the full name) at MS2 level. Matched oxonium fragments y and b ions can be seen which indicate the O-glycosylation at serine 259. Oxonium fragment ions with high peak intensities make the identification of the glycopeptide sequence challenging. The attached O-glycan was HexNAcHexNeuAc here. Cysteines are carbamidomethylated.

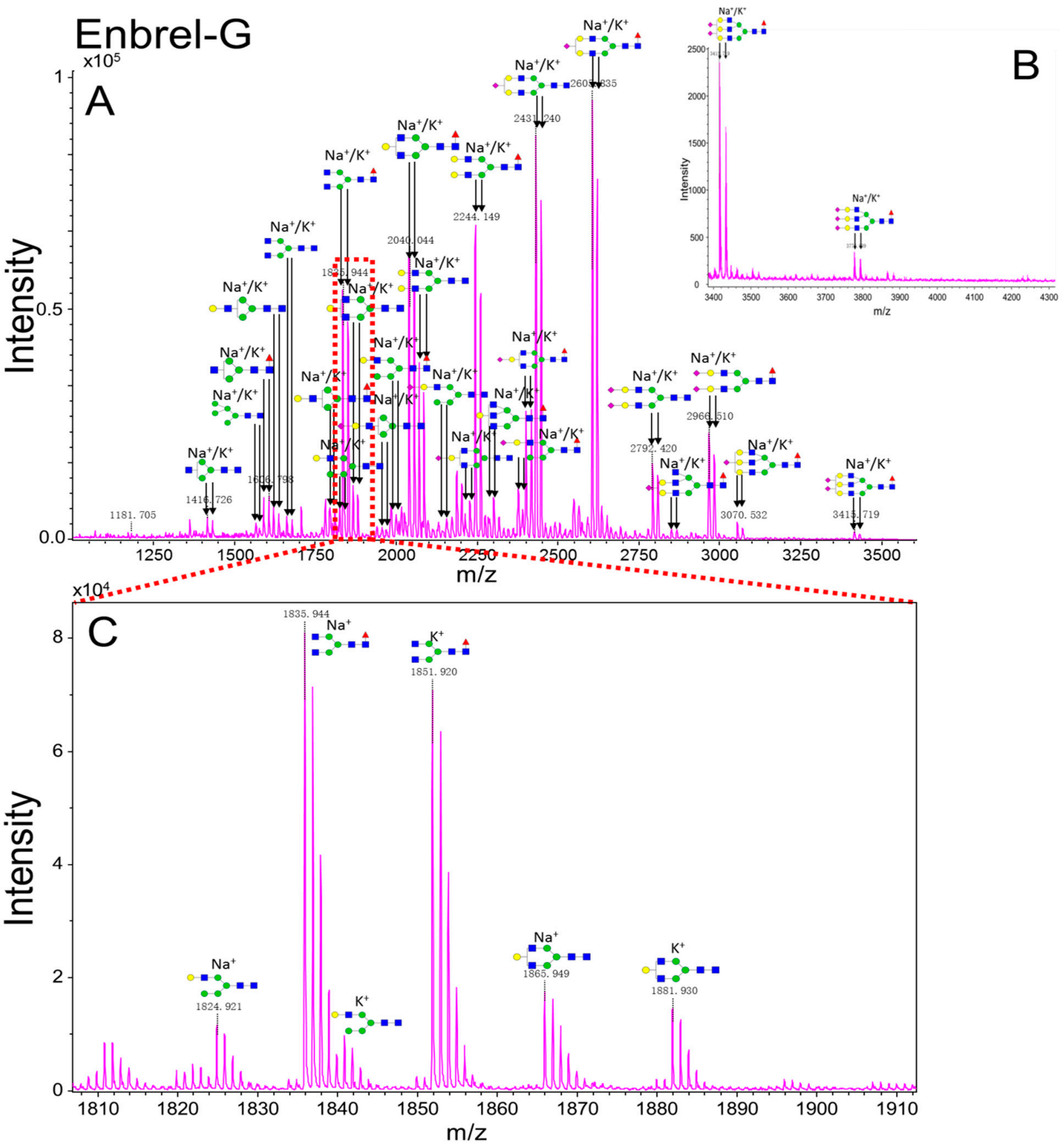

Figure S6: MALDI-MS mass spectrum of permethylated N-glycans released from Enbrel-G. Mass spectrum A and B were the measurement of global permethylated N-glycans, in which each N-glycan was seen with sodium and potassium adduct as one pair; Mass spectrum C is the enlarged region representing the area from *m/z* 1810 to *m/z* 1910. The signals correspond to $GlcNAc_3Man_4Gal_1$, $GlcNAc_4Man_3Fuc_1$ and $GlcNAc_4Man_3Gal_1$ N-glycans.

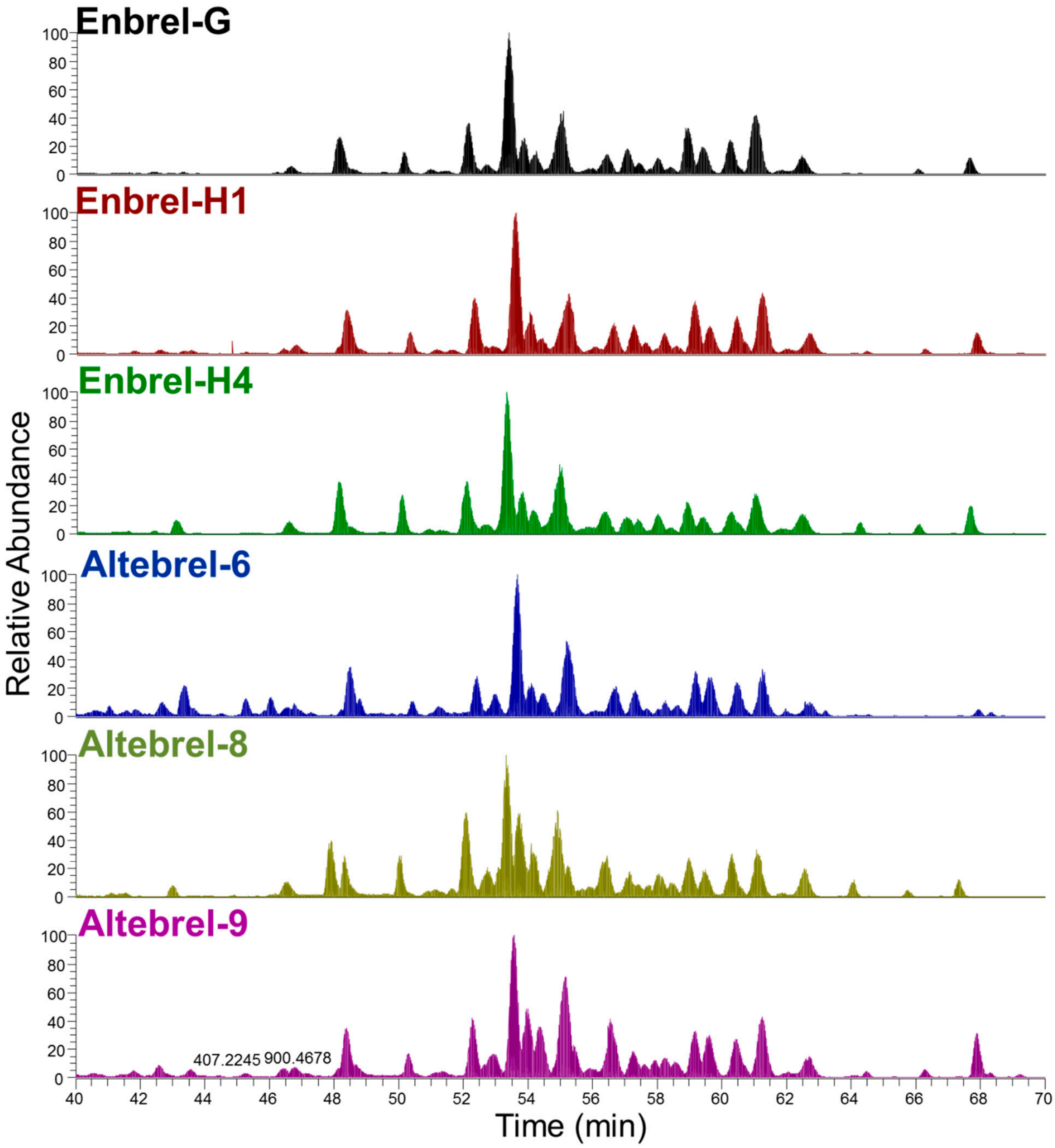

Figure S7: BPCs of permethylated N-glycans derived from different etanercept samples from two vendors.

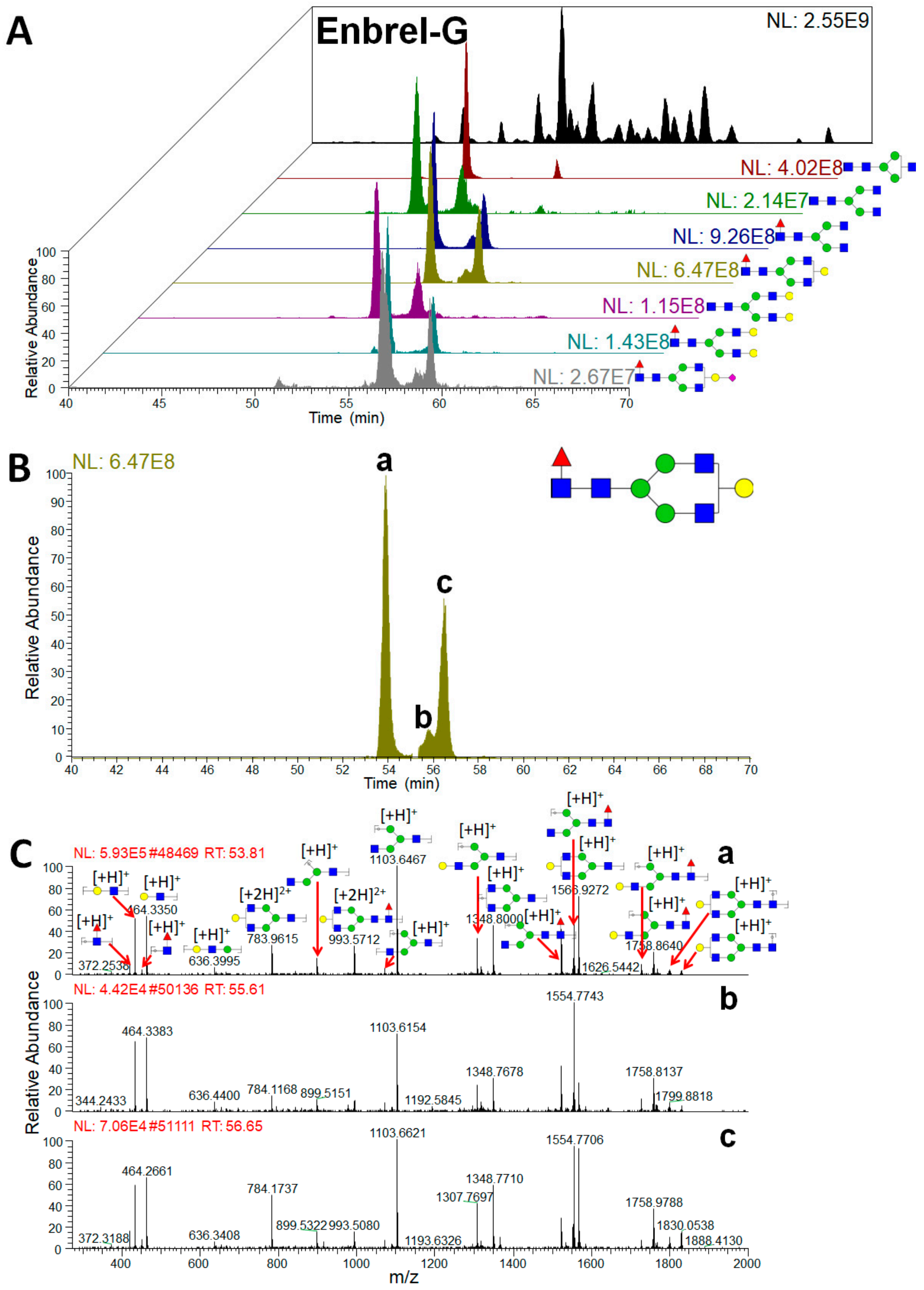

Figure S8: Chromatograms and spectra from N-glycans from Enbrel-G representing different isomers. (A) XICs of several isomeric N-glycans showed different retention times which were related to the size of the N-glycan structures, attached fucose and sialic acid content. (B) XIC of GlcNAc3Man3Gal1Fuc1 showed different isomeric structures as a, b and c. (C) The detailed fragment illustration for each structure of the N-glycan composition as GlcNAc3Man3Gal1Fuc1. The fragment spectra in parts b and c have the same annotation with the part a, therefore all signals belong to the same fragments.

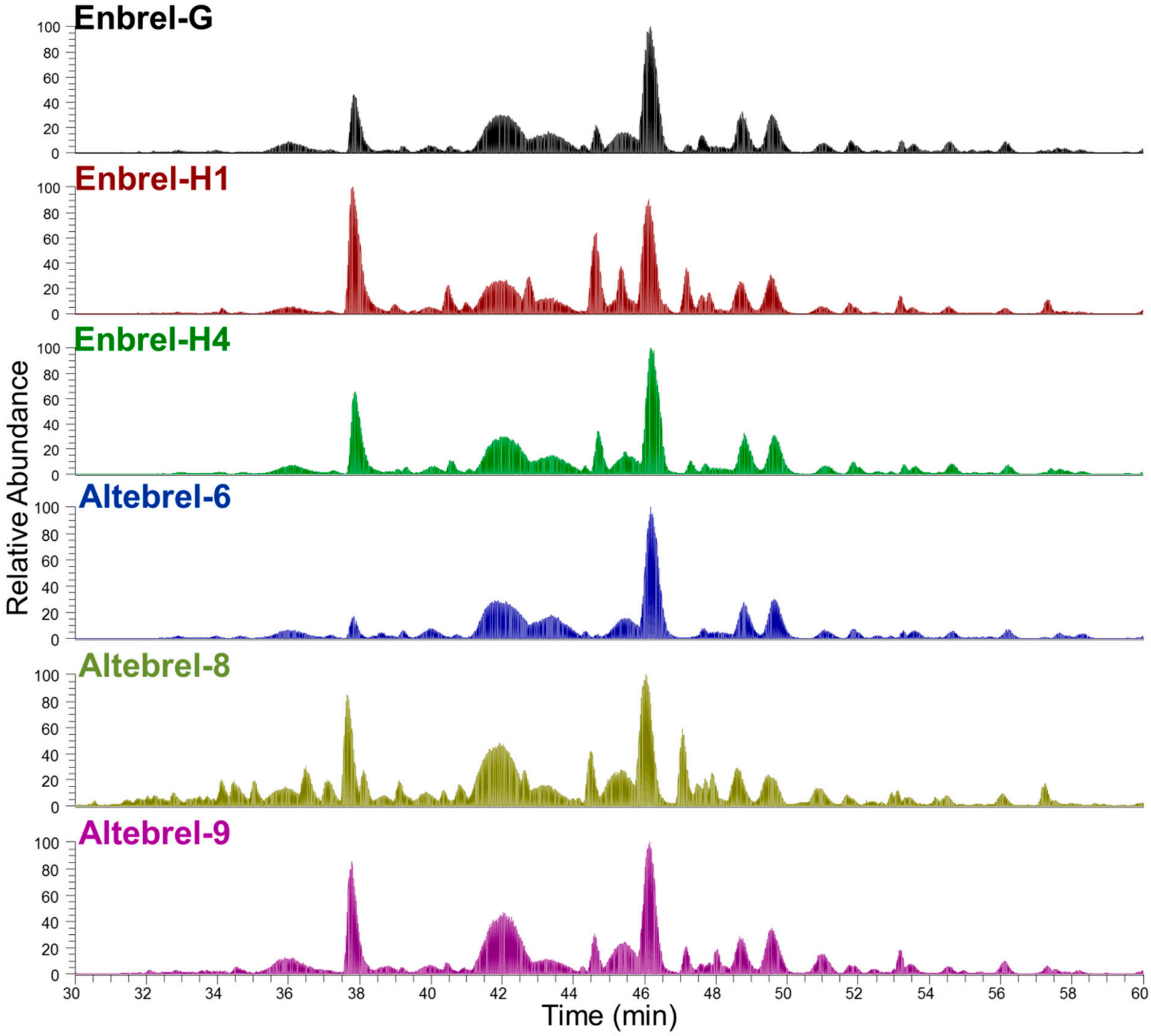

Figure S9: BPCs of O-glycans from different vendors.

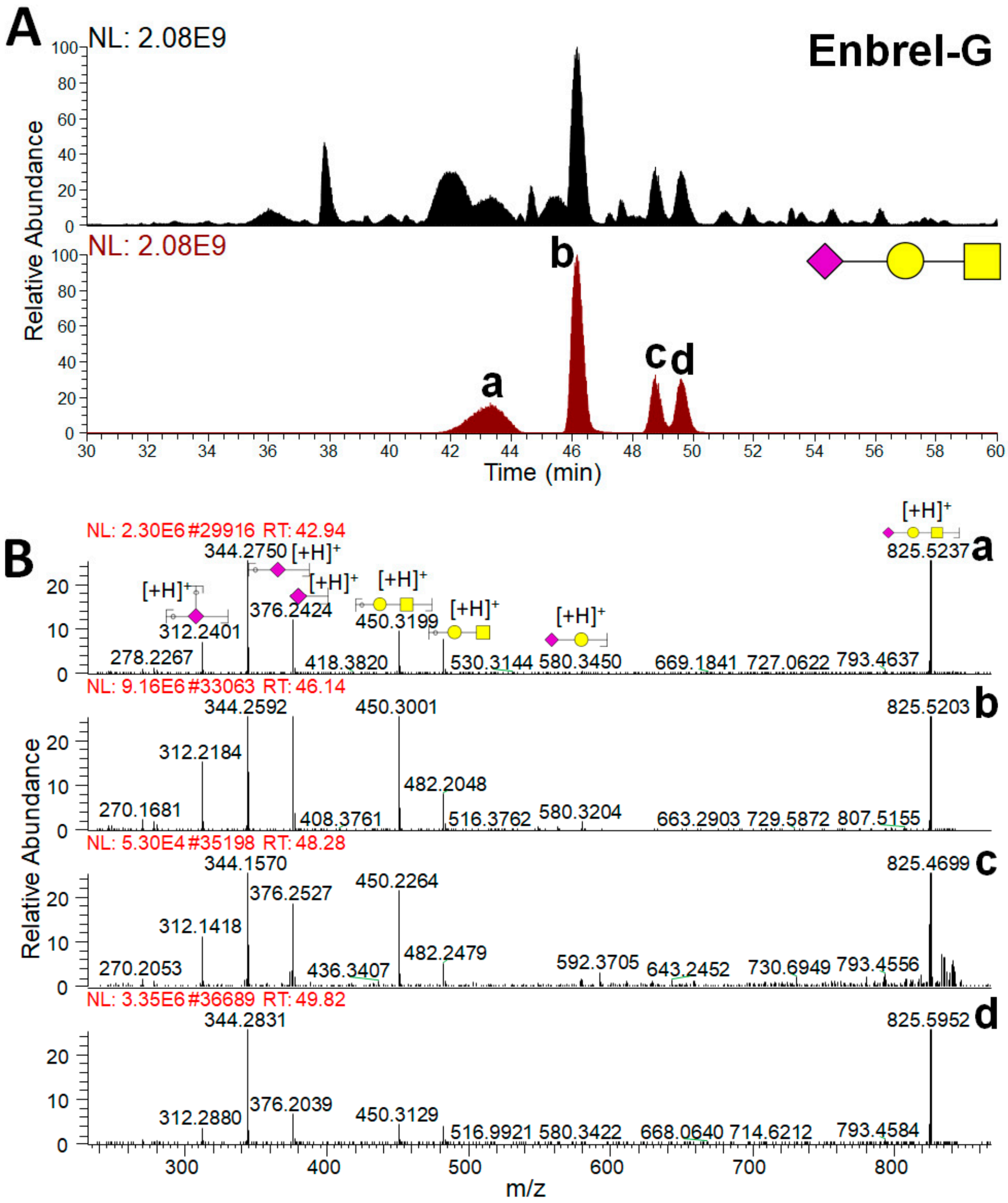

Figure S10: Chromatograms and spectra of O-glycans from Enbrel-G showing different isomers. (A) XIC of GalNAc1Gal1NeuAc1 for the isomeric structures as a, b, c and d. (B) Fragment spectra of isomers of the O-glycan (GalNAc1Gal1NeuAc1). The fragment ions at *m/z* 450.3199 and 580.3450 in the MS2 spectrum a and *m/z* 482.2048 in MS2 spectrum b confirmed the linear structure; above fragments also existed in spectra c and d with the same annotation.

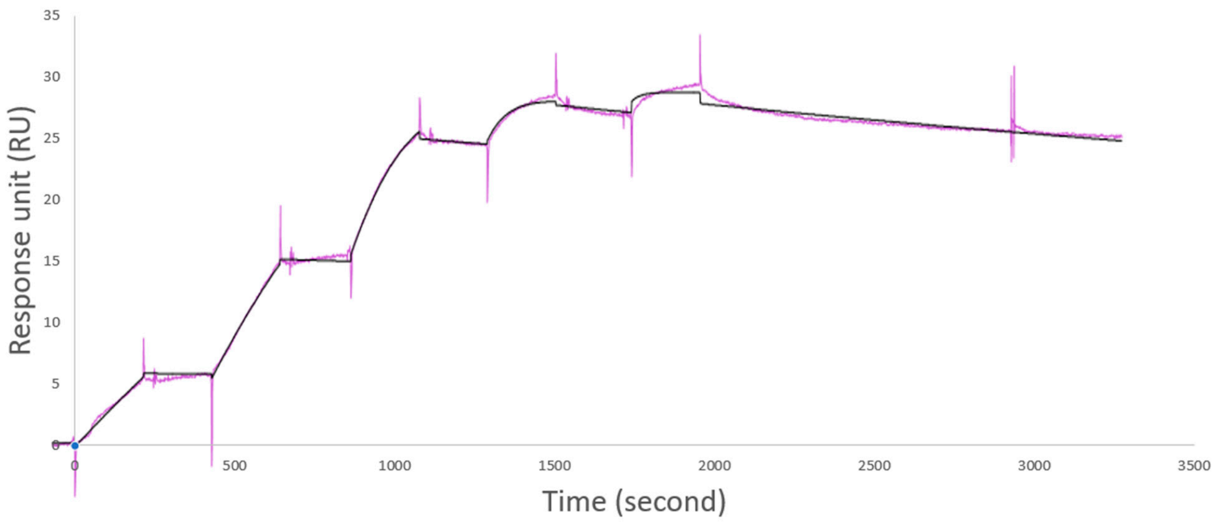

Figure S11: Single cycle kinetics graph of Altebrel-8 with the fitted model. In the association phase, five increasing concentrations of TNFα pass the chip surface which contains captured etanercept by anti-human IgG antibody. This results in an increasing level of RU. In the dissociation phase, HBS-EP buffer will pass and detach the bound TNFα from etanercept.

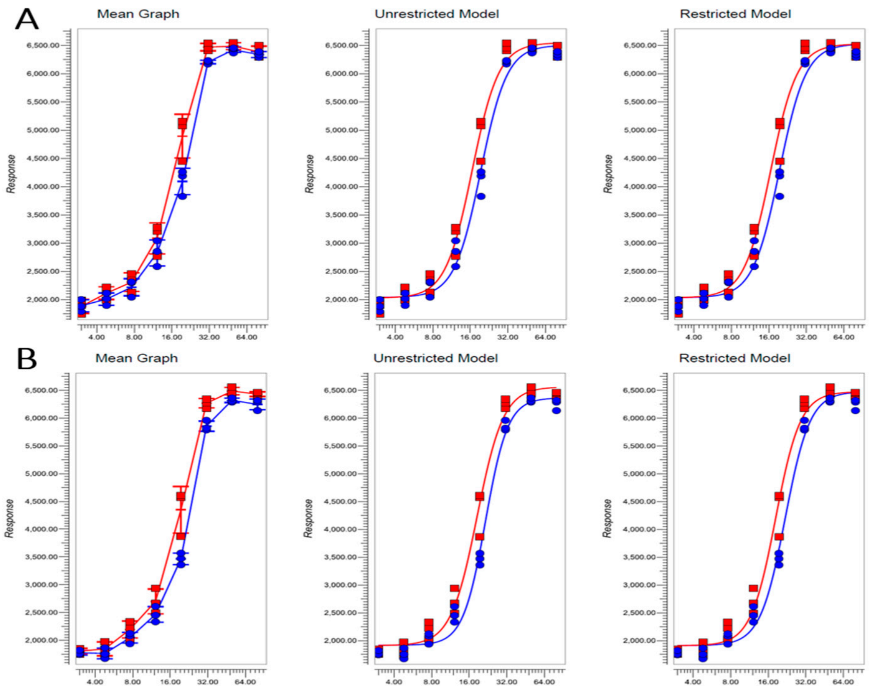

Figure S12: Dose-response curves of A) Control Enbrel-H1 and B) Control Altebrel-8 versus the in-house reference standard.

Table S1: List of observed modifications in etanercept. Modified amino acids are bolded and underlined in the sequence. Lack of lysine and leucine in C-terminal and N-terminal ends are shown by (–)

Modification	Modified amino acid	Mass shift	Exemplary peptide
**Formation of pyroglutamic acid**	Glu	-18.0106	**E**PQVYTLPPSR
**Dioxidation**	Trp	+31.9898	FN**W**YVDGVEVHNAK
**Oxidation**	Met	+15.9949	DTL**M**ISR
**Carbamylation**	Lys, Met, Peptide N-terminus	+43.0058	EEMT**K**NQVSLTCLVK
**Amidation**	Asp, Glu	-0.984	TPEVTCVVVDVSHEDP**E**VK
**Deamidation**	Asn, Gln	+0.984	GFYPSDIAVEWES**N**GQPENNYK
**C-terminal Lysine clipping**	Lack of Lys	-128.09497	SLSLSPG(–)
**N-terminal heterogeneity**	Lack of Leu	-113.08407	(–)PAQVAFTPYAPEPGSTCR

Table S2: Identified N-glycans in Enbrel-G.

**Reference**	***N*-glycan structures**	**Native Monoisotopic Mw**	**Permethylation Monoisotopic Mw**		**Deviation (ppm)**
			**Experiment**	**Theory**	
Confirmation of N-glycans previously reported [6]	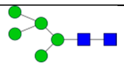	1234.4334	1556.7920	1556.7945	-1.6059
	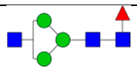	1259.4651	1567.8078	1567.8094	-1.0205
	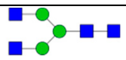	1316.4865	1638.8456	1638.8476	-1.2204
	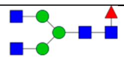	1462.5444	1812.9346	1812.9369	-1.2687
	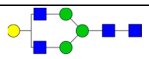	1478.5394	1842.9446	1842.9476	-1.6278
	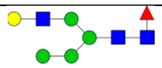	1583.5707	1976.0073	1976.0089	-0.7996
	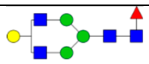	1624.5973	2017.0356	2017.0369	-0.6445
	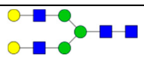	1640.5922	2047.0442	2047.0475	-1.6121
	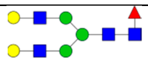	1786.6501	2221.1334	2221.1368	-1.5308
	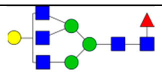	1827.6767	2262.1607	2262.1618	-0.4686
	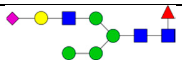	1874.6661	2337.1811	2337.1826	-0.6204

Table S2. *Cont.*

	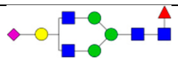	1915.6927	2378.2074	2378.2108	-1.4297
	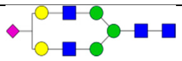	1931.6876	2408.2192	2408.2214	-0.9135
	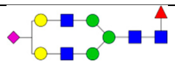	2077.7455	2582.3084	2582.3107	-0.8907
	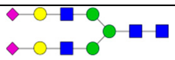	2222.7830	2769.3916	2769.3953	-1.3360
	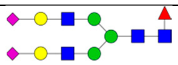	2368.8409	2943.4812	2943.4846	-1.1551
	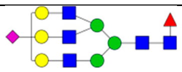	2442.8777	3031.536	3031.5371	-0.3629
Newly identified N-glycans	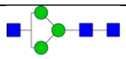	1113.4072	1393.7196	1393.7202	-0.4305
	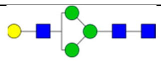	1275.4600	1597.8182	1597.8199	-1.0639
	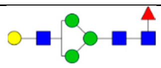	1421.5179	1771.9084	1771.9104	-1.1287
	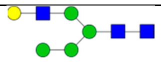	1437.5128	1801.9191	1801.9197	-0.3163
	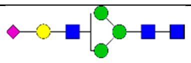	1566.5554	1958.9924	1958.9936	-0.6126
	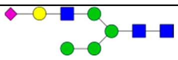	1728.6082	2163.0913	2163.0934	-0.9431
	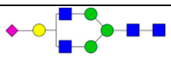	1769.6348	2204.1178	2204.1215	-1.6788
	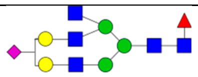	2280.8249	2827.4334	2827.4352	-0.6366
	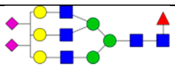	2733.9731	3392.7062	3392.7062	-1.4148
	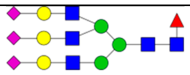	3025.0686	3753.8814	3753.8824	-0.9057
